# Development of Ammonia Selectively Permeable Zeolite Membrane for Sensor in Sewer System

**DOI:** 10.3390/membranes11050348

**Published:** 2021-05-10

**Authors:** Hisao Inami, Chie Abe, Yasuhisa Hasegawa

**Affiliations:** 1 Hitachi Ltd., Research & Development Group, 832-2 Horiguchi, Hitachinaka 312-0034, Japan; hisao.inami.w@hitachi.com; 2 National Institute of Advanced Industrial Science and Technology (AIST), Research Institute for Chemical Process Technology, 4-2-1 Nigatake, Sendai 983-8551, Japan; abe-chie@aist.go.jp

**Keywords:** zeolite membrane, ammonia, hydrogen sulfide, separation, sensor

## Abstract

Ammonia (NH_3_) and hydrogen sulfide (H_2_S) are hazardous and odorous gases. A special device that is not affected by other gases is necessary so that it can detect such gases. Zeolite membranes can separate the desired component selectively by molecular sieving and selective adsorption. LTA-, MFI-, and FAU-type zeolite membranes were prepared in this study, and the permeation and separation performances were determined for the ternary mixture of NH_3_, H_2_S, and N_2_ to develop an NH_3_ selectively permeable membrane. Although the separation factors of NH_3_ were high enough for the LTA-type zeolite membrane, the NH_3_ permeance was the lowest among the three membranes. In contrast, the FAU-type zeolite membrane with Si/Al = 1.35 showed a high enough NH_3_ permeance and a NH_3_/N_2_ separation factor. The membrane modification and varying the membrane composition were carried out to reduce the H_2_S permeance. As a result, the H_2_S permeance could be decreased by modification with silane coupling agents, and a separation factor of NH_3_ toward H_2_S of over 3000 was achieved.

## 1. Introduction

Ammonia (NH_3_) and hydrogen sulfide (H_2_S) occur in sewers, waste treatment plants, oil and gas wells, and volcanoes, naturally. These gases in sewers lead to several serious problems, such as health hazards for workers and the corrosion of concrete and metals. Therefore, in Japan, the concentrations of NH_3_ and H_2_S in working environments are limited to below 25 ppm and 1 ppm, respectively. Their emission regulations are lower (1 ppm for NH_3_ and 0.02 ppm for H_2_S) [1]. In particular, the emission regulation of H_2_S is significantly higher than the threshold of the human sense of smell (0.0004 ppm). Therefore, sensitive NH_3_ and H_2_S sensors are important to manage the concentrations of NH_3_ and H_2_S in sewer systems.

Constant potential electrolytic gas sensors are widely used in the commercial field. The sensors have two electrodes, the working electrode and the reference electrode, and the potential between these electrodes is kept constant. The gas concentration can be determined by the electrolysis of gases at the working electrode. However, it is difficult for this method to distinguish the target gas from similar gases. Unique mechanisms, where the target gas flows into the detecting chamber selectively, are necessary to detect the target gas selectively [2].

Zeolites are aluminosilicate compounds with a regular-sized micropore structure, and the micropore diameter is similar to the size of gas molecules. A polycrystalline zeolite layer can separate the target component from mixtures. LTA-type zeolite membranes have been used for the dehydration of biomass-derived ethanol [3]. Much research on this topic has been reported since the 1990s, although a large zeolite membrane is needed for use in chemical processes, such as dehydration [4]. In contrast, there is little research on small devices, such as sensors. For example, Coronas et al. developed a sensor equipped with an LTA-type zeolite membrane [5], and the sensor can detect ethanol selectively, apart from coexistent CH_4_.

The permeation and separation performance of zeolite membranes are explained by the surface diffusion mechanism [6,7,8,9,10]. Molecules adsorbed on the pore wall of zeolite diffuse into the pores with the concentration difference between both the sides of the membrane as the driving force in the mechanism. Moulijn and coworkers [6,7,8,9,10] proposed that the Maxwell–Stefan diffusion formula can be used to describe the gas permeation properties of MFI-type zeolite membranes.

In this study, to develop a unique NH_3_ sensor, NH_3_ selective zeolite membranes were studied. The development goals were to achieve separation factors of NH_3_ toward N_2_ and H_2_S of 100. First, LTA, MFI, and FAU-type zeolite membranes were prepared on an α-Al_2_O_3_ support tube by a hydrothermal process, and the permeation and separation performances of the membranes were determined for diluted NH_3_ and H_2_S mixtures. Furthermore, the effects of ion exchange and the surface modification of the zeolite membrane on the permeation and separation properties are also discussed in this paper.

## 2. Experimental

### 2.1. Membrane Preparation

#### 2.1.1. LTA-Type Zeolite Membrane

An LTA-type zeolite membrane with Si/Al = 1 was prepared on the outer surface of a porous α-Al_2_O_3_ tube by the secondary growth of seed crystallites [11]. Sodium aluminate (Wako pure Chemicals Industry) was dissolved in distilled water in a beaker, and a sodium silicate solution (Nakarai) was dissolved in distilled water in the other beaker. The solution containing the aluminum source was added to the silicon source solution slowly, and the mixture was stirred at room temperature for 1 h. The molar composition of the solution was 2 SiO_2_: 1 Al_2_O_3_: 2 Na_2_O: 100 H_2_O. The porous α-Al_2_O_3_ tube was used as the support in this study, and the properties were as follows: outer diameter = 2.0 mm, inner diameter = 1.5 mm, porosity = ca. 40%, and mean pore diameter = 150 nm. The outer surface of the support tube was rubbed with LTA-type zeolite particles (Wako Pure Chemicals Industry) to implant seeds for nucleation. The tube was added to a Teflon-lined stainless-steel autoclave filled with 30 g of the synthesis solution, and the autoclave was placed in the oven at 373 K for 4.5 h horizontally to form a polycrystalline LTA-type zeolite layer on the support tube. After the reaction, the support tube was recovered, washed with distilled water, and dried in air overnight at room temperature to obtain the LTA-type zeolite membrane (hereafter referred to as the LTA membrane).

#### 2.1.2. MFI-Type Zeolite Membrane

An all-silica MFI-type zeolite membrane was prepared on the outer surface of the porous α-Al_2_O_3_ tube [12]. Sodium hydroxide (Wako pure Chemicals Industry), colloidal silica (JGC C&C, Cataloid SI-30), and tetrapropylammonium bromide (TPABr, Tokyo Chemicals Industry) was dissolved in distilled water, and the clear solution was stirred at room temperature for 1 h. The molar composition of the solution was 1 SiO_2_: 0.05 Na_2_O: 0.1 TPABr: 80 H_2_O. The outer surface of the support tube was rubbed with MFI-type zeolite particles to implant seeds for nucleation, and the tube was added to the autoclave filled with 30 g of the synthesis solution. The autoclave was placed in the autoclave at 413 K for 24 h horizontally to form a polycrystalline MFI-type zeolite layer on the support. After the reaction, the support tube was recovered, washed with distilled water, and dried overnight at room temperature. Finally, the membrane was calcined in air at 673 K for 48 h to remove the organic structure-directing agent to obtain the MFI-type zeolite membrane (hereafter referred to as the MFI membrane).

#### 2.1.3. FAU-Type Zeolite Membranes

FAU-type zeolite membranes with different Si/Al ratios were prepared on the outer surface of the porous α-Al_2_O_3_ support tubes by a hydrothermal process [13]. Sodium hydroxide and sodium aluminate were dissolved into distilled water in a beaker. A sodium silicate solution was dissolved in distilled water in the other beaker. The solution containing the aluminum source was added to the silicon source solution, and the solution was stirred at room temperature for 4 h. The molar composition of the solution was *x* SiO_2_: 1 Al_2_O_3_: 1.3*x* Na_2_O: 75*x* H_2_O (*x* = 5 and 25). The FAU-type zeolite membrane with the different molar composition will be referred to as the FAU-*x* membrane, hereafter. *x* and *x* SiO_2_ are the same as *x* and FAU-*x*.

The outer surface of the support tube was rubbed with NaY-type zeolite particles (Toso Corp., HSZ-320NAA) to implant seeds for nucleation, and the tube was added to the autoclave filled with 30 g of the synthesis solution. The autoclave was placed in an oven at 363 K for 20 h to form a polycrystalline FAU-type zeolite layer on the support tube. After the reaction, the support tube was recovered, washed with distilled water, and dried in air overnight at room temperature to obtain the FAU-type zeolite membrane (hereafter referred to as the FAU-x membrane).

The FAU-5 membrane was rinsed in an aqueous solution containing either RbCl or CsCl for the ion-exchange. After heating at 333 K for 3 h, the membrane was washed with distilled water and dried overnight in air at room temperature to obtain the Rb- and Cs-exchanged FAU-type zeolite membranes.

### 2.2. Modification with Silane Coupling Agents

**Figure 1:**
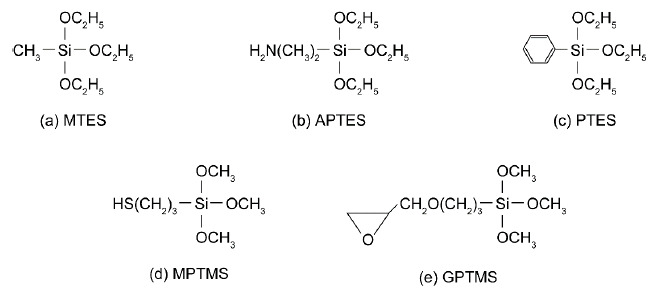
Silicon compounds using modification of FAU-type zeolite membrane in this study. (**a**) Methyltriethoxysilane (MTES), (**b**) 3-aminopropyltriethoxysilane (APTES), (**c**) phenyltriethoxysilane (PTES), (**d**) 3-mercaptopropyltrimethoxysilane (MPTMS), and (**e**) 3-glycidoxypropyltrimethoxysilane (GPTMS).

The FAU-5 membrane was modified with either methyltriethoxysilane (MTES, Shin-Etsu Silicone), 3-aminopropyltriethoxysilane (APTES, Shin-Etsu Silicone), phenyltriethoxysilane (PTES, Shin-Etsu Silicone), 3-mercaptopropyltrimethoxysilane (MPTMS, Shin-Etsu Silicone), or 3-glycidoxypropyltrimethoxysilane (GPTMS, Shin-Etsu Silicone), as shown in Figure 1. The silane coupling agent was diluted with toluene, and the concentration was 5 wt%. The FAU-5 membrane with the length of 40 mm was added to the solution. The solution was heated at 343–413 K for 1–20 h for the modification of the FAU-5 membrane. The membrane was washed with acetone and dried overnight at room temperature to obtain silane-modified membrane.

Silane modified FAU-type zeolite particles were also prepared under the same conditions as those for the membrane to determine the amount of the modified silane coupling agent.

### 2.3. Characterization

The crystal structure of the membrane was determined by X-ray diffraction (XRD, Rigaku, Smart Lab.), and the morphology was observed using a scanning electron microscope (SEM, Hitachi high technologies, TM-1000). The composition of the zeolite layer was analyzed by an energy-dispersive X-ray (EDX) analyzer attached with the SEM in order to determine the Si/Al ratio of zeolites and degree of ion-exchange. The degree of ion-exchange was calculated from the decreased amount of Na+ in the zeolite. The amount of modified silane compounds was determined using thermogravimetry (TG-DTA, Rigaku Thermo Plus).

### 2.4. Gas Permeation Test

**Figure 2:**
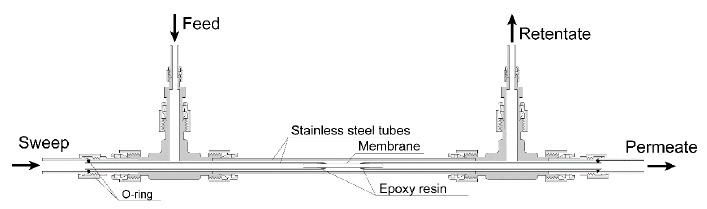
Schematic illustration of the permeation cell.

The gas permeation properties of the zeolite membranes were determined using a sweep gas method [14]. Both ends of the membrane were connected to stainless steel tubes with resin (Varian, Torr Seal), and the effective membrane area for permeation was ca. 1.6 cm^2^. The membrane was fixed to a permeation cell, as shown in Figure 2. The ternary gas mixture (NH_3_: H_2_S: N_2_ = 0.05: 0.05: 99.9 vol%) was fed onto the outer surface of the membrane (feed side) at 20 mL min^−1^, and helium was introduced into the inside of the membrane (permeate side) at 1.0 mL min^−1^ as the sweep gas. The total pressures of both sides of the membrane were kept at an atmospheric pressure. The gas compositions were analyzed using a mass spectrometer (Hiden Analytical), and the gas flow rate was determined by a bubble flow meter. The permeances was calculated as follows [14]:

(1) $$(\mathrm{permeance})=\frac{\left(\mathrm{transferred}\;\mathrm{mole}\;\mathrm{per}\;\mathrm{unit}\;\mathrm{time}\right)}{\left(\mathrm{membrane}\;\mathrm{area})(\mathrm{partial}\;\mathrm{pressure}\;\mathrm{difference}\right)},$$

The separation factor was defined as the ratio of permeances in this study [14].

## 3. Results and Discussion

### 3.1. Membrane Characterization

**Figure 3:**
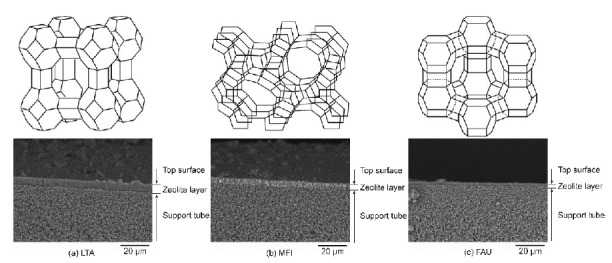
Schematic illustration of zeolite structures and SEM images of fractured sections of (**a**) LTA-, (**b**) MFI-, and (**c**) FAU-type zeolite membranes.

**Figure 4:**
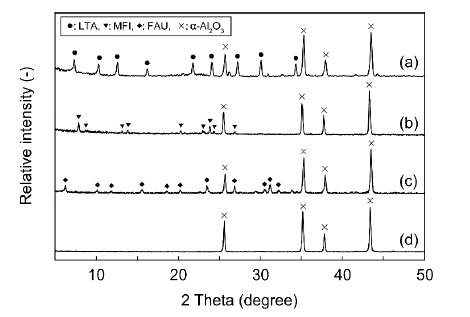
XRD patterns of (**a**) LTA-, (**b**) MFI-, (**c**) FAU-type zeolite membranes, and (**d**) support tube.

Figure 3 shows the SEM images of the zeolite membranes prepared in this study. The outer surface of the support tube was completely covered with a polycrystalline layer for all membranes, and the thicknesses of the LTA, MFI, and FAU-5 membranes were ca. 6 μm, 4 μm, and 2 μm, respectively. The XRD patterns of the membranes contained peaks of both corresponding zeolite and α-Al_2_O_3_, as shown in Figure 4. These results suggest that the LTA-, MFI-, and FAU-type zeolite membranes preparation on the porous α-Al_2_O_3_ support tube succeeded.

The EDX measurement data showed that the Si/Al ratios of the FAU-5 and FAU-25 membranes were 1.35 and 1.67, respectively. Furthermore, the ion-exchange degrees of Rb and Cs for the FAU-5 membranes were 75% and 60%, respectively. Assuming that the composition of the synthesis solution is *x* SiO_2_: 1 Al_2_O_3_: 1.3*x* Na_2_O: 75*x* H_2_O, the theoretical Si/Al ratio of FAU-type zeolite can be calculated as follows [15]:

(2) $$\mathrm{Si}/\mathrm{Al}=\frac{2.3x-1}{1.3x-1}.$$

Based on this equation, the Si/Al ratio of the zeolite layer is estimated to be 1.40 and 1.69 at *x* = 5 and 25, respectively. The experimental Si/Al ratio of 1.35 and 1.67 at *x* = 5 and 25 agreed with the theoretical values. Furthermore, the maximum degrees of ion exchange of Rb^+^ and Cs^+^ are 78 and 64% for NaX-type zeolite, respectively [16]. These results suggest that the FAU-type zeolite membranes with the desired Si/Al ratios were prepared and the ion exchange succeeded.

**Figure 5:**
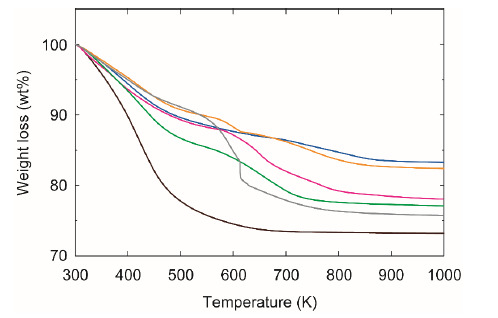
TG curves of non-modified (black), MTES-modified (blue), APTES-modified (red), PTES-modified (green), MPTMS-modified (orange), and GPTMS-modified (gray) FAU-type zeolite particles.

Figure 5 shows the TG curves of FAU-type zeolite particles modified with silane coupling agents. The particles modified with silane coupling agents showed a weight loss below 523 K, and additional weight losses were also observed at 523–873 K. The weight loss below 523 K was due to the water desorption of zeolite, and that at 523–873 K was attributed to the oxidative decomposition of silane coupling agents and the water desorption. The weight loss due to the oxidative decomposition can be ignored for the particles with no modification. The weight losses for the non-modified particles were 23.3 wt% below 523 K and 3.4 wt% at 523–873 K. The weight loss due to the water desorption in the high temperature range is estimated to be 14.6% of that in the low temperature range. Based on this relation, we estimated the amount of modified silane compounds by subtracting the amount of water desorption in the high temperature range.

Table 1 summarizes the modification conditions and the amount of modified silane compounds. The amount of modified silane increased with temperature and reaction time. For example, the amount of MTES was 0.09 mol kg^−1^ at 373 K for 20 h, and that increased to 0.31 mol kg^−1^ with the temperature being increased to 413 K. For PTES, moreover, the modification amount was 0.02 mol kg^−1^ for 1 h, increased with time, and reached 0.35 mol kg^−1^ for 20 h. It is considered that the difference in the modification amount is due to the reactivity of silanes.

The weight losses of the modified particles due to water desorption are smaller than those of the non-modified particles, as shown in Figure 5. This suggests that the silane coupling agents bonded to the oxygen atoms inside the cavity, not the oxygen atoms on the outer surface of the particles. Assuming that the number of oxygen atoms per unit area and the specific surface area of FAU-type zeolite are 10^19^ atoms m^−2^ and 700 m^2^ g^−1^, respectively [17], the maximum amount of the modified silane coupling agent is estimated to be ca. 12 mol kg^−1^. However, the maximum modification amount was 0.587 mol kg^−1^ for GPTMS in our experiment. Since the diameter of the cavity of FAU-type zeolite is 1.3 nm [16], the narrow space of the zeolite micropores was attributed to the lower modification amount.

### 3.2. Gas Permeation Properties

#### 3.2.1. Effect of Zeolite Framework Structure

**Figure 6:**
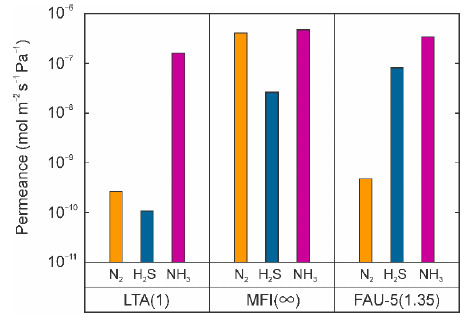
Effect of crystal structure of zeolite on gas permeation properties of membranes for ternary mixtures of NH_3_, H_2_S, and N_2_ at 295 K.

Figure 6 shows the effect of the zeolite framework on the gas permeation properties for the ternary gas mixture of NH_3_, H_2_S, and N_2_ at 295 K. The NH_3_ permeances for all the membranes were the highest among N_2_, H_2_S, and NH_3_, while the N_2_ and H_2_S permeances were affected by the zeolite framework structure. For the LTA membrane, the separation factors of NH_3_ toward N_2_ and H_2_S were 600 and 1500, respectively, and the membrane showed the NH_3_ selective permeation properties. These separation factors were high enough and favorable for NH_3_ sensors. However, the NH_3_ permeance of the membrane was the lowest among the three membranes (1.6 × 10^−7^ mol m^−2^ s^−1^ Pa^−1^). Since low NH_3_ permeance leads to reduction of the response rate, this membrane is not suitable for the NH_3_ sensor. For the MFI membrane, the NH_3_ permeance was three times higher than that of the LTA membrane. However, the separation factors of NH_3_ toward N_2_ and H_2_S were 1.2 and 18, respectively. The NH_3_ permeance of the FAU-5 membrane was 3.4 × 10^−7^ mol m^−2^ s^−1^ Pa^−1^, and this was slightly lower than that of the MFI-type zeolite membrane due to the strong adsorption of NH_3_ on FAU-type zeolite. The separation factors of NH_3_ toward N_2_ and H_2_S were 710 and 4.2, respectively. The NH_3_ permeance and the separation factor toward N_2_ were enough for the NH_3_ sensor. Therefore, we selected the FAU-type zeolite membrane as the candidate for an NH_3_ selective membrane.

The highest separation factor of the FAU-type zeolite membrane is attributed to the selective adsorption of NH_3_ due to the coulomb interaction. The permeation and separation properties of zeolite membranes are explained by the surface diffusion mechanism, where the molecules adsorbed on an adsorption site moved to neighbor adsorption sites [18]. NH_3_ and H_2_S, which have intermolecular partial charges, interact with zeolites with cations strongly [19]. As a result, these molecules block the zeolite micropores, and it becomes difficult for the weaker adsorption component, such as N_2_, to penetrate the micropores [20]. Furthermore, the relation between the sizes of molecules and zeolite micropores is also important for molecular sieving. The molecular sizes of N_2_, H_2_S, and NH_3_ are 0.364, 0.36, and 0.26 nm, respectively [16]. The micropore diameters of the LTA-, MFI-, and FAU-type zeolite are ca. 0.4 nm, 0.51–0.56 nm, and 0.74 nm, respectively [21].

Since NH_3_ was the smallest and most strongly adsorbed molecule in our experiments, the NH_3_ permeances were the highest for all the membranes. The adsorbed NH_3_ molecules filled the micropores of the LTA-type zeolite and inhibited the permeation of N_2_ and H_2_S molecules, the sizes of which were similar to the micropore diameter. As a result, the LTA membrane showed high separation factors of NH_3_. The micropore diameter of the FAU-type zeolite is larger than that of the LTA-type zeolite, although NH_3_ molecules are adsorbed strongly. Therefore, the NH_3_ molecules could not fill the micropore completely. This resulted in the N_2_ and H_2_S permeances being larger than those of the LTA membrane.

#### 3.2.2. Effect of Zeolite Composition

The H_2_S permeance of the FAU-5 membrane was the highest among the three membranes, as shown in Figure 6. For the development of the NH_3_ selective membrane, it is important to reduce the H_2_S permeance. Here, we determined the effects of the zeolite composition on the permeation and separation performances of the FAU-type zeolite membrane.

**Figure 7:**
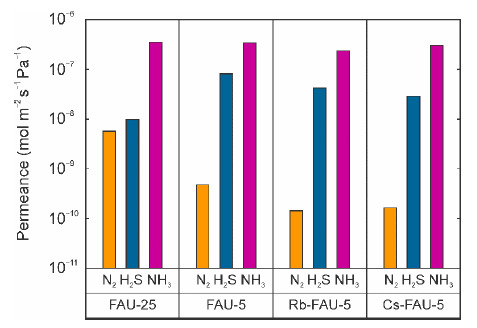
Effect of zeolite composition on gas permeation properties of FAU-type zeolite membranes for ternary mixtures of NH_3_, H_2_S, and N_2_ at 295 K.

Figure 7 shows the effects of the Si/Al ratio and cation species on the gas permeation properties for the ternary gas mixture at 295 K. The NH_3_ permeances of the membranes were (2.4–3.5) × 10^−7^ mol m^−2^ s^−1^ Pa^−1^ and were independent of the Si/Al ratio and cation species. The N_2_ permeance of the FAU-25 membrane with Si/Al = 1.67 was higher than that of the FAU-5 membrane with Si/Al = 1.35. In contrast, the permeance of H_2_S showed the reverse tendency since the number of strong adsorption sites decreased with reducing the Al content. The N_2_ and H_2_S permeances were decreased slightly by the ion exchange with Rb^+^ and Cs^+^. The micropore diameter of FAU-type zeolite was reduced from 0.7 nm to 0.6 nm by the ion exchange of Na^+^ with K^+^ and Rb^+^ [22]. The lower N_2_ and H_2_S permeances were due to the reduction in the micropore diameter by ion exchange.

The effects of the zeolite composition on the permeation properties were determined for the reduction of the H_2_S permeance in this section. However, the effect of the composition on the permeation properties was lower than that of the framework structure. These results suggest that a significant change in the affinity and/or micropore diameter of zeolite is necessary to improve the separation performance.

#### 3.2.3. Effect of Modification with Silane Coupling Agents

**Figure 8:**
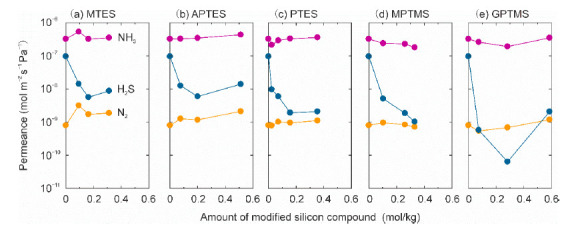
Effect of modification with silicon compounds on permeances of FAU-type zeolite membranes for ternary mixtures of NH_3_, H_2_S, and N_2_ at 295 K. (**a**) MTES, (**b**) APTES, (**c**) PTES, (**d**) MPTMS, and (**e**) GPTMS.

**Figure 9:**
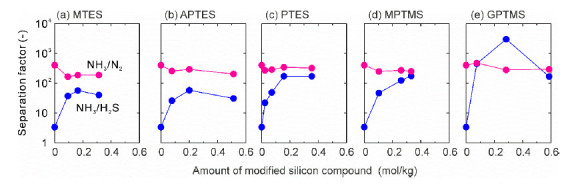
Influence of modification with silicon compounds on the separation factors of FAU-type zeolite membranes for ternary mixtures of NH_3_, H_2_S, and N_2_ at 295 K. (**a**) MTES, (**b**) APTES, (**c**) PTES, (**d**) MPTMS, and (**e**) GPTMS.

The FAU-5 membrane was modified using silane coupling agents, as shown in Figure 1, to change the affinity and micropore diameter of zeolite. Figure 8 and Figure 9 show the effect of modification with the silicon compounds on the permeances and separation factors for the ternary gas mixture at 295 K. The NH_3_ permeances were (1.8–5.4) × 10^−7^ mol m^−2^ s^−1^ Pa^−1^ and independent of the modification agents and the amount of modified silicon compounds. The N_2_ permeances are also constant around 10^−9^ mol m^−2^ s^−1^ Pa^−1^. For all the membranes, the separation factors of NH_3_ toward N_2_ were higher than 100. In contrast, the H_2_S permeances were decreased largely for all kinds of silane coupling agents. The H_2_S permeance decreased with the increasing amount of modification and reached the minimum at 0.15–0.30 mol kg^−1^.

The minimum H_2_S permeances were (5–6) × 10^−9^ mol m^−2^ s^−1^ Pa^−1^ for MTES and APTES, (1–2) × 10^−9^ mol m^−2^ s^−1^ Pa^−1^ for PTES and MPTMS, and 6 × 10^−11^ mol m^−2^ s^−1^ Pa^−1^ for GPTMS. As a result, the separation factor of NH_3_ toward H_2_S achieved the target value 100. In particular, for GPTMS, the separation factor increased over 3000. This result suggests that the silicon compounds blocked the pathway of H_2_S molecules selectively.

As discussed in relation to Figure 5, the silane coupling agents bonded to the oxygen atoms inside the cavity of the FAU-type zeolite. The agents located in the cavity could not block the permeation of NH_3_ molecules and only blocked the permeation of H_2_S molecules. Furthermore, the constant N_2_ permeances mean that the modification with silanes does not influence the micropore diameter of FAU-type zeolite and membrane thickness. In addition, N_2_ molecules may pass through the small intercrystalline boundaries of the membrane. We conclude that it is effective for an NH_3_ selective membrane to modify the cavity of zeolite using silane coupling agents.

## 4. Conclusions

The effects of the zeolite framework structure, composition, and modification with silicon compounds on the permeation and separation performance of the membranes were determined for the ternary mixture of NH_3_, H_2_S, and N_2_ in this study to develop an NH_3_-selective membrane. As a result, the LTA-type zeolite membrane showed high separation factors of NH_3_, and the FAU-type zeolite membrane with high Al content is promising among the zeolite membranes used in this study. Furthermore, the ion exchange and surface modification of the membrane were carried out for the reduction in H_2_S permeance in this study. The H_2_S permeance could be decreased significantly by modification with GPTMS. These results suggest that the separation factor of NH_3_ can be increased by the modification of zeolite membranes.

## Figures and Tables

**Table 1:** Modification condition and modification amount of silicon compound.

Compound	Modification Condition		Amount of Modification (mol kg^−1^)
	Temp. (K)	Time (h)	
MTES	373	20	0.09
	413	5	0.16
	413	20	0.31
APTES	313	18	0.08
	373	3	0.20
	413	5	0.51
PTES	413	1	0.02
	413	3	0.07
	413	5	0.16
	413	20	0.35
MPTMS	413	1	0.10
	413	3	0.26
	413	20	0.33
GPTMS	343	3	0.08
	373	3	0.28
	413	5	0.59

## Data Availability

Not applicable.
